# A First Implementation of Underwater Communications in Raw Water Using the 433 MHz Frequency Combined with a Bowtie Antenna

**DOI:** 10.3390/s19081813

**Published:** 2019-04-16

**Authors:** Samuel Ryecroft, Andrew Shaw, Paul Fergus, Patryk Kot, Khalid Hashim, Adam Moody, Laura Conway

**Affiliations:** 1 Built Environment and Sustainable Technologies (BEST) Research Institute, Liverpool John Moores University, Liverpool L3 3AF, UK; S.P.Ryecroft@2012.ljmu.ac.uk (S.R.); P.Fergus@ljmu.ac.uk (P.F.); P.Kot@ljmu.ac.uk (P.K.); K.S.Hashim@ljmu.ac.uk (K.H.); 2 United Utilities, Warrington WA5 3LP, UK; Adam.Moody@uuplc.co.uk (A.M.); Laura.Conroy@uuplc.co.uk (L.C.)

**Keywords:** Bowtie Antenna, Under Water Wireless Sensor Network, Underwater Communication, Sensor Networks, Water Pollutants, Water Quality

## Abstract

In 2016, there were 317 serious water pollution incidents in the UK, with 78,000 locations where businesses discharge controlled quantities of pollutants into rivers; therefore, continuous monitoring is vital. Since 1998, the environment agency has taken over 50 million water samples for water quality monitoring. The Internet of Things has grown phenomenally in recent years, reaching all aspects of our lives, many of these connected devices use wireless sensor networks to relay data to internet-connected nodes, where data can be processed, analyzed and consumed. However, Underwater wireless communications rely mainly on alternative communication methods such as optical and acoustic, with radio frequencies being an under-exploited method. This research presents real world results conducted in the Leeds and Liverpool Canal for the novel use of the 433 MHz radio frequency combined with a bowtie antenna in underwater communications in raw water, achieving distances of 7 m at 1.2 kbps and 5 m at 25 kbps.

## 1. Introduction

Water quality is tightly monitored and regulated in the UK, with legislation covering both drinking water [1] and natural water resources such as rivers, lakes and reservoirs [2]. In 2016, 86% of river water bodies had not reached a good ecological status, with the leading reason being agriculture, rural land management, water industry and urban and transport pressures. In 2016, there were a total of 317 serious water pollution incidents in the UK, with water industry being responsible for 60 incidents [3]. In the UK, 55% of water abstracted from freshwater was for use in the public water supply [4]. Current monitoring approaches rely on manual collection of samples with processing taking part in a centralized lab location, this leaves gaps between samples collection where areas are not monitored. There is a need for a continuous monitoring approach to provide a better technique for reducing the dependency on manual sample collection.

The proposition of an alternative communication method for Underwater Wireless Sensor Networks (UWSNs), could have an impact on areas such as environmental monitoring, combining developments in sensing technologies for substances, including nitrates [5], heavy metals and bacteria [6], carbonyl compounds [7] and dissolved oxygen [8]. Other areas such as industry, including offshore oil and renewable energy, wave energy harvesting, and defense, such as costal monitoring, as well as water utility companies could benefit from the proposed monitoring technique.

Wireless communications underpin many modern technologies that have become pervasive in modern life, from mobile phones to home wireless networks. The same communication technologies also underpin the operation of many internet-connected devices, forming part of the IoT (Internet of Things) and the Industrial Internet of Things (IIoT). The IoT market worldwide is predicted to reach a value of $457.29 billion by 2020 [9]. With applications of IoT technologies being applied in a range of industries from smart energy meters to intelligent agriculture. While the adoption of IoT in conventional environments has thrived, there has been little development in IoT applications in underwater environments, with one of the issues being underwater wireless communications being non-trivial.

If an effective communication method in (Underwater Wireless Sensor Networks (UWSNs) were achieved, applications could include a network of water quality monitoring sensors connected through an UWSN to a wider internet connection or private network to allow for the development of a smart, continuous water quality monitoring system. Such a system could enable remote monitoring of key water quality metrics and allow for the automation of processes within water treatment works to adjust to incoming water properties, and in cases where water quality is unsuitable for processing, an automated shutoff of the water intake.

Many aspects of WSNS can be transferred and applied to UWSNs, and considerations such as security [10,11], energy-efficient routing approaches [12,13,14], and application layer protocols [15,16] have all been considered in detail with a vast amount of coverage in existing works. Little work has been carried out into the area of underwater wireless communication methods, for which Wireless Sensor Networks (WSNs) and UWSNs differ, with WSNs primarily using Radio Frequency (RF), and UWSNs tending to use acoustic and optical communications.

At present, RF communication is not widely used in a UWSNs; instead, methods such as acoustic and optical are used. Factors impacting UWSNs are highlighted in research conducted by both X. Che et al. [17] and S. Ryecroft et al. [18]. Some research has explored using RF at other frequencies in underwater environments, such as the work conducted by Lloret et al. [19], which achieved communication at 20 cm at up to 11 Mbps using a frequency of 2.4 GHz. Rubino et al. [20] examined the use of a frequency of 868.3 MHz and FSK (Frequency Shift Keying) to transmit compressed images. The experimental work was undertaken in a controlled environment using a water pool. The proposed research was able to achieve data rates of 4.9 KB/s at distances of up to 5 m with some packet loss. Other research undertaken by A. Shaw et al. [21] investigated the use of lower frequencies using a loop antenna design; the results achieved distances of over 90m using frequencies of 3.5 MHz, 4.7 MHz and 5 MHz with the results being achieved by combining a loop antenna with a directional design, meaning that alignment between the two antennas is required. These results were also achieved using a highly tuned antenna that would not function outside of water.

Previous research by A. A. Abdou et al. [22,23] explored using the 433 MHz frequency to communicate in an underwater environment, and this work showed that a 433 MHz signal could be transmitted and detected at distances of up to 30 m using bowtie antennas. The work made use of a bowtie antenna, also known as a Bifin antenna, to communicate at distances of over 30 m. Bowtie antennas are a simplified form of a log-periodic toothed planar antennas. Bowtie antennas are the planar version of the finite biconical antenna. Bowtie antennas have a bidirectional pattern, with broad main beams perpendicular to the plane of the antenna, and are linearly polarized [24]. Work by A. Shaw et al. [21] investigated the use of loop antennas, which are highly directional, making them ideal for the formation of point to point links, but being of little use in underwater sensor networks in long term deployments.

Underwater wireless sensor networks currently utilize two communication methods, optical modulates a light source, which is then detected by a photodiode. The second communication approach commonly used is acoustic communication, which encodes data through the modulation of a sound wave. Optical communication can offer significant data rates of up to 500 Mbps at distances of up to 150 m using commercial products such as the Bluecomm 5000 series [25]. Optical provides a point-to-point link, which requires alignment between two nodes, communication speeds and distances can be impacted by the alignment as well as other factors such as optical clarity of water and turbidity.

Commercial acoustic communication devices such as the Teledyne marine 900 series [26] offer data rates from 80 bps up to 15,360 bps, depending on configuration and encoding methods, at operating distances of 2 km to 6 km. Acoustic communication offers the ability to implement a broadcast topology unlike optical, and investigations undertaken by Codarin, A et al. [27] and research carried out by Peng, C et al. [28] have both highlighted issues surrounding the introduction of noise into a marine environment, including impacting predator–prey balances, damaging hearing of marine wildlife, and disrupting reproductive cycles. Acoustic communications also suffer when operating in noisy environments, such as in offshore drilling, where noise poses an issue for acoustic communications. Acoustic communications also suffer when deployed in shallow water, with issues such as reverberation impacting on data rates and distances.

RF communication can offer higher data rates. The Nyquist theorem [29] states that if the rate of an original signal transmission is 2B, then a signal with frequencies no greater than B is sufficient to carry the signal rate. In cases of binary data, the maximum transmission rate can be calculated using the calculation of B Hz is 2B bps. It is possible to achieve higher data rates using a multi-level transmission in which case the calculation becomes *2B log_2_ M.* In non-perfect environments, the maximum data rate that can be achieved in a given communication channel is constrained by factors such as signal-to-noise ratio. The Shannon-Hartley Theorem [30] combines the work of Hartley [31], which provides calculations for the amount of information per second that can be encoded into a given bandwidth. Shannon-Hartley builds on this work by including signal-to-noise ratio, allowing Equation (1) to be derived:

(1) $$C=B\;log_{2}\left(1+SNR\right)$$

*C* = channel capacity (bits per second)
*B* = Bandwidth of the channel (Hz)
*SNR* = Signal-to-noise ratio.

In addition to increased data rates, RF communication also offers advantages such as a reduced setup complexity of the network due to there being no need for alignment when using an omnidirectional antenna design. RF communication is also unaffected by factors such as water turbidity, unlike optical communications. RF communication is also immune to the impacts of environmental noise and is not impacted by shallow waters, unlike acoustic communications, which can suffer from the reverberation of transmitted signals impacting the achievable data rates or even stopping communication completely. RF communication combined with an omnidirectional antenna design such as the bowtie antenna enables an easier setup process that supports a mesh network topology. RF communication also offers superior data rates to those offered by acoustic communications. RF communication can cross the water air boundary allowing for data transmission between submerged network nodes and non-submerged network nodes. The design of the bowtie antenna demonstrated in this research also allows for communication in both air and water due to the ultra-wide band design.

## 2. Materials and Methods

Initial lab-based experimentation was undertaken in a controlled environment set to establish the impact that the conductivity of water would have on the attenuation of the RF signal as it passed through the water. Experiments were conducted in a small container with antennas separated at set intervals. A HopeRF 69 HW transceiver module produced by Shenzen HOPE Microelectronics Co, Shenzen, China combined with an Atmel 328p micro controller, Microchip Technology, Chandler, USA were used to produce a continuous signal at 433 MHz, a Hameg HMS 3000 spectrum Analyzer, manufactured by Hameg instruments, Mainhausen, Germany was used to measure the received signal strength. A pair of matched Bowtie antennas designed for use with the 433 MHz frequency were used throughout the experimental work, each antenna was coated with a layer of clear epoxy resin to protect the antenna surface and stop conduction across the two poles of the antenna.

**Figure 1:**
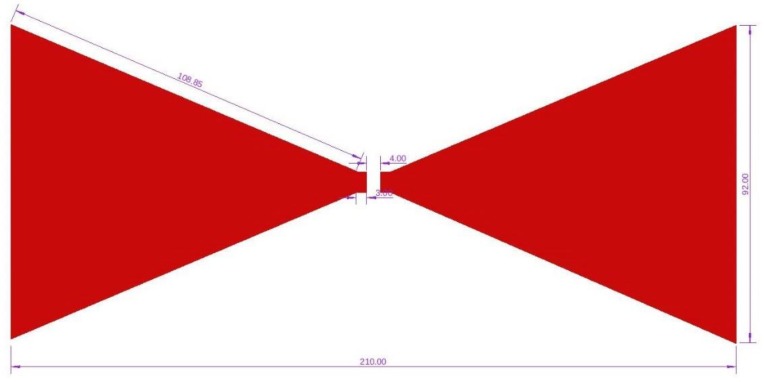
Bowtie antenna design with dimensions in mm.

The bowtie antennas used throughout experiments were designed in Autodesk Eagle created by Auto Desk, San Rafael, USA and printed using a chemical etching process onto FR4 PCB Produced by C.I.F, Buc, France with substrate with a thickness of 1.5 mm and a copper thickness of 35 µm. A layer of epoxy resin was applied to the exposed copper face of the antenna, with a thickness of approximately 0.8 mm. The design of the antenna and the dimensions (in mm) used are shown in Figure 1. The bowtie antenna used was based on designs used in previous research undertaken by A. A. Abdou et al. [22,23] using the 433 MHz frequency.

The water within the tank had an initial conductivity of 0.2 mS/cm, the conductivity in the tank was raised using sea salts. The conductivity of a 1 molar solution was used to calculate the amount of sea salts required to raise the conductivity by the desired amount, the correct weight of salts was then added to the water tank and the tank agitated to dissolve the salts. Conductivity readings were then taken with a Hanna Instruments 98188 conductivity meter at each corner and the center of the water taken to ensure that conductivity of the water was correct across the whole tank. The conductivity was raised to 0.4 mS/cm, 0.6 mS/cm, 0.8 mS/cm, 1 mS/cm and 5.2 mS/cm; by comparison, normal levels of conductivity in drinking water are limited by legislation to 0.25 mS/cm and seawater has a conductivity of approximately 40 mS/cm.

The two antennas were suspended and separated at distances between 10 cm and 50 cm, inclusive, with readings taken at 5 cm intervals. The transmitting antenna remained stationary while the receiving antenna was moved further away. Once moved the antennas were left for settle for at least one minute to ensure that the signal stabilized. A reading was taken from the spectrum analyzer for each position the antenna was placed in before the antenna was moved to the next position. Readings were taken 10 times for each position at each conductivity level.

After the completion of lab-based work initial field tested was undertaken at the end of the Liverpool Canal. Testing was undertaken in this environment to examine how signals would propagate and carry in a real-world environment over larger areas and distances. The field trials used a microcontroller combined with a HopeRF69 HW transceiver produced by Shenzen HOPE Microelectronics Co, Shenzen, China; the hardware was connected to a temperature and depth sensor to collect data that could be transmitted from one node to another, and the experiment collected the received signal strength. The sensor nodes were deployed at varying distances along the canal edge, and the sensor node was left in place for +/− 1 min to allow the water to settle before readings for 10 transmissions were taken. Once readings had been taken, the receiving node was moved to the next reading location and redeployed. The distances and average readings can be seen in Table 1.

Experiments were undertaken using On-Off Keying (OOK) modulation at two baud rates: 1.2 kbps and 25 kbps to allow for a comparison of how baud rates impact on the communication distances that can be achieved. OOK modulation encodes digital data using either the presence of a frequency or not to encode binary data. OOK modulation was chosen due to the simplicity of operation and does not depend on factors such as frequency or phase of the carrier wave.

During the field testing, the temperature of the water was measured using a TSYS01 temperature sensor manufactured by TE connectivity, Schaffhausen, Switzerland and was averaged as 6.1 °C. The conductivity of the water was measured as 0.51 mS/cm using a Hanna Instruments HI933000 conductivity meter manufactured by Hanna Instruments, Woonsocket, USA.

For each baud rate an estimated maximum distance was established by moving the receiving antenna until data was no longer received, these maximum distances provide the furthest distance tested. The receiving antenna was placed at 1 m separation intervals and left to settle for 1 min before a Received signal strength indication (RSSI) reading was recorded. Each reading was taken on three separate occasions and an average taken, the results for both baud rates are provided in Table 1.

**Figure 2:**
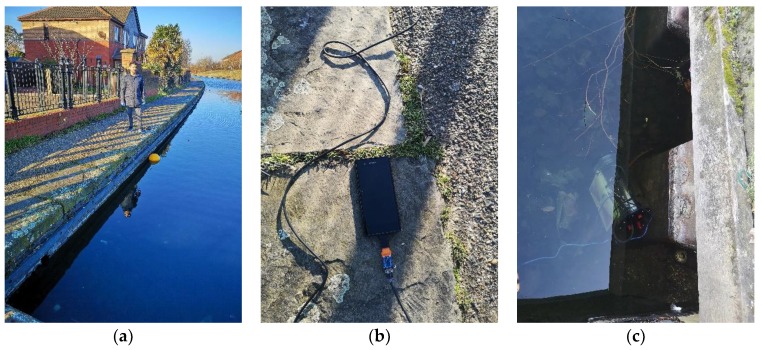
(**a**) experimental setup; (**b**) Data capture device; (**c**) Submerged sensor node.

Figure 2 shows images of the experimental field setup and site, Figure 2a shows the setup with the two communication nodes deployed, Figure 2b shows the connection of the receiving node to an android smart phone using a serial adapter to capture result, Figure 2c shows the submerged transmitting node.

## 3. Results and Discussion

The results from the lab-based experiments provided in Table 2 follow the established principle that the conductivity of the water through which the signal passes have an impact on signal loss. The signal loss was particularly noticeable in water with a conductivity of 5.2 mS/cm, with significant additional losses being observed. It is likely that these initial experiments were based on near field power rather than far field power. Near-field power is normally observed at distances of up to 1 wavelength and decays much faster than far field power.

**Figure 3:**
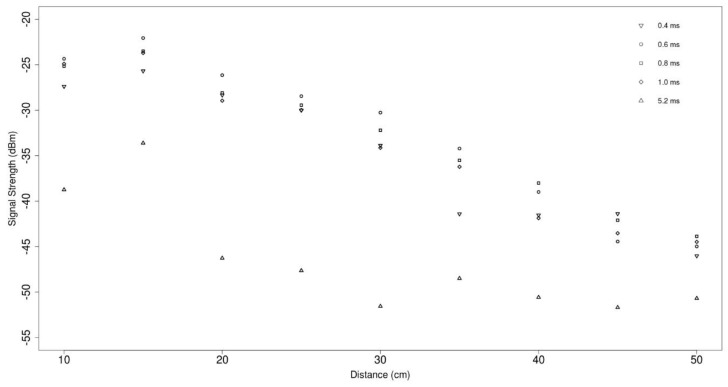
A graph showing the signal loss across distances with a range of conductivities.

Figure 3 shows a plot of the averaged data collected from the lab-based experimentation. The standard deviation between the ten readings for each conductivity and distance combination, in all cases the standard deviation showed that all results fell within +/− 3 dBm and in most cases fell within +/− 1 dBm.

The hardware platform developed for further experimentation had a transmission power of 14 dBm and was capable of receiving signals at signal power of −120 dBm. It is however, important to consider losses that will take place due to coupling both on the transmitter and the receiver, making it likely that the power transmitted will be lower.

Field-based work identified that the baud rate used during communication had a direct impact on the communication ranges that were achievable in real world environments with higher baud rates having a negative impact on the achievable communication distances. This is due to the weaker signal being harder to distinguish from the background RF noise. Slower baud rates improve the ability of the receiver to distinguish the transmission from the spectral noise. The maximum communication rate of a given channel can be calculated using the Shannon-Hartley Therom, which provides a theoretical maximum bitrate that can be achieved over a given channel given the bandwidth, signal power and signal-to-noise ratio.

The results from the field work can be seen in Table 1, showing the distance tested and the average received RSSI for each for the two baud rates tested as well as a combined average where applicable.

The results in Table 1 shows that signal degradation is similar between the two baud rates, which is to be expected, as baud rate does not impact on the signal itself, but rather the ability to distinguish the signal from background noise. The results follow the principle of the Shannon-Hartley theorem that state as a received signal strength weakens the baud rate must be lowered to allow for the signal to be distinguished from the background noise.

The results provided in Table 1 present an average of the data collected during field work based on three separate deployments of the sensor node, the standard deviation of the data was calculated, across baud and distance combinations using the three data points collected. The standard deviation showed that in most cases, there was a standard deviation of 5 dBm and with the remaining readings having a standard deviation of no more than 5.7 dBm.

The results show that the reduction of baud rate allows an improved range to be achieved, with an increase of 40% being achieved. This suggests that the signal-to-noise ratio past 5 m makes reliable higher baud rate communication impossible, but still allows lower baud rate communications extend the communication distance further. These results indicate that lower baud rates could further increase transmission ranges.

The results indicated that the distances achieved could be extended in two ways to allow for greater communication distances. The first would be to identify a more powerful transmitter or amplify the output signal of the current transited module. Amplifying the signal may increase the amount of noise within the signal, which may in turn impact of the reliability of the encoding of data used. Since OOK depends on the presence or lack of presence of a frequency, it is likely that amplification would have less of an impact on the encoded data than other methods available. Alternatively, a further reduction on baud rate could further increase the communication range achievable at the expense of data rates. It is likely that a combination of these two approaches would yield significantly improved ranges by providing a stronger signal to begin with as well as improving the ease of identification of the signal from background noise.

The experimental work indicated that a baud rate of 25 kbps could be used to achieve communication at distances of 5 m in real world conditions. This provides the option of high-speed shorter-range wireless communication that could be applied to a range of applications such as the transmission of images and short-range control of UAVs.

The field work indicated that the slower baud rate of 1.2 kbps could be used to achieve communication at distances of up to 8 m providing a range of opportunities in a variety of fields such as in the development of a multimode underwater environmental monitoring system for areas such as reservoirs. Such a system would not require significant data rates, but demanding reasonable communication distances to reduce the node density required to cover large monitoring areas.

The results indicate that a more in-depth protocol and wider supporting architecture targeted at the 433 MHz spectrum could be developed that includes a form of link speed negotiation existing wireless protocols such as Wi-Fi support link speed negotiation known as the physical layer rate. Such a protocol could use lower data rates to identify nearby network nodes and begin a link speed negotiation process with nodes adjusting data rates until a suitable data rate is achieved. Such a mechanism could be used for a variety of applications in areas such as continuous environmental monitoring.

Existing research has already shown that frequencies such as 868.3 MHz can be used in controlled environment to communicate data at comparable distances, the results from Rubino et al. establish an effective communication rate of 4.9KB/s using the 868.3 MHz frequency, compared to the maximum data rate examined in this study of 25 kbps equivalent to 3.125 KB/s, it is possible that future research could further investigate increasing the data rates.

Other existing research such as Lloret et al. achieved significantly higher data rates of 11 mbps, although at heavily reduced distances of 20 cm.

Existing solutions within optical underwater communication such as the bluecom 100 series offers data rates of 5 mbps at distances of up to 5 m. The obtained results offer reduced data rates compared to this optical communication method, but provides slightly improved distances at lower data rates than mentioned product. In addition, proposed technique overcome existing limitations with the optical communication.

Other solutions such as acoustic communication the Teledyne marine 900 series offers significantly better ranges than the results obtained with communication distances of between 2 km and 6 km; however, data rates achieved during field work show significantly better data rates, with baud rates of between 140 bps to 15,360 bps being offered by the Teledyne marine 900, compared to the baud rates of 1200 bps and 25000 bps achieved during experimental work.

It is possible for transmission distances to be improved further with alterations to the circuitry being made to incorporate a higher power transmitter and a higher sensitivity receiver module; however, short-range communications do have applications in localized sensor networks, where a high density of nodes may be desirable. With commercial products offering link distances of 5 m and data rates as low as 140 bps, it is clear there is a gap within the underwater communication area for a solution that provides short communication distances at medium communication speeds with the benefit of allowing a mesh topology with no need of alignment. The novelty of this research is to deploy a distributed sensor network in shallow water for water security.

## 4. Conclusions

The results demonstrated that RF communications is achievable in a real-world environment at distances of 5 m with data rates of 25 kbs and at distances of 7 m with a data rate 1.2 kbps, indicating that RF communications can be used to communicate data in an underwater environment, which could be used as the basis of forming an underwater wireless sensor network, with scope for improving communication distances in a variety of ways in future research.

The research presents a novel application of 433 MHz communication in an underwater environment using a bowtie antenna specifically designed to communicate in underwater environments at a range of conductivities as well as in the air. The research findings are supported through field trials in real world conditions with a varying conductivity of the water.

## Figures and Tables

**Table 1:** Results from field work with averages for the 1.2 kbps, 25 kbps and the overall average, where applicable.

Distance (m)	1.2 kbps Average (dBm)	25 kbps Average (dBm)	Overall Average (dBm)	Standard Deviation 1.2 kbps (dBm)	Standard Deviation 25 kbps (dBm)
1	−49.00	−46.00	−47.50	5.71547607	2.692582
2	−69.33	−72.00	−70.67	3.8586123	3.674235
3	−87.33	−88.33	−87.83	3.68178701	3.832427
4	−87.67	−94.00	−90.83	1.24721913	5.612486
5	−107.00	−106.33	−106.67	2.1602469	1.581139
6	−109.67	N.A	N.A	1.24721913	N.A
7	−110.33	N.A	N.A	0.47140452	N.A

**Table 2:** Signal losses for distances between 10 cm and 50cm at different conductivity levels.

Distance (cm)	0.4 mS/cm (dBm)	0.6 mS/cm (dBm)	0.8 mS/cm (dBm)	1.0 mS/cm (dBm)	5.2 mS/cm (dBm)
10	−27.38	−24.34	−25.15	−24.9	−38.74
15	−25.675	−22.06	−23.5	−23.7	−33.61
20	−28.33	−26.14	−28.09	−28.95	−46.27
25	−30	−28.45	−29.44	−29.97	−47.64
30	−33.87	−30.26	−32.2	−34.13	−51.57
35	−41.4	−34.21	−35.51	−36.22	−48.5
40	−41.527	−38.99	−38.01	−41.87	−50.58
45	−41.38	−44.44	−42.1	−43.53	−51.7
50	−46.02	−44.98	−43.87	−44.48	−50.69

## References

[1] HM Government (2016). The Water Supply (Water Qaulity) Regulations 2016.

[2] HM Government (2017). Water Resources, England and Wales. The Water Environment (Water Framework Directive) (England and Wales) Regulations.

[3] Environment Agency (2018). The State of the Environment: Water Quality.

[4] Environment Agency (2018). The State of the Enviroment Water Resources.

[5] Cashman S., Korostynska O., Shaw A., Lisboa P., Conroy L. (2017). Detecting the Presence and Concentration of Nitrate in Water Using Microwave Spectroscopy. IEEE Sens. J..

[6] Clausen H.C., Dimaki M., Bertelsen V.C., Skands E.G., Rodriguez-Trujillo R., Thomsen D.J., Svendsen E.W. (2018). Bacteria Detection and Differentiation Using Impedance Flow Cytometry. Sensors.

[7] Cheng J., Shao J., Ye Y., Zhao Y., Huang C., Wang L., Li M. (2018). Microfluidic Preconcentration Chip with Self-Assembled Chemical Modified Surface for Trace Carbonyl Compounds Detection. Sensors.

[8] Zhang Y., Angelidaki I. (2012). A simple and rapid method for monitoring dissolved oxygen in water with a submersible microbial fuel cell (SBMFC). Biosens. Bioelectron..

[9] (2017). Market Pulse Report Internet of Things (IoT).

[10] Pathan A.S.K., Hyung-Woo L., Choong Seon H. Security in wireless sensor networks: Issues and challenges. Proceedings of the 8th International Conference Advanced Communication Technology.

[11] Hari P.B., Singh S.N. Security issues in Wireless Sensor Networks: Current research and challenges. Proceedings of the International Conference on Advances in Computing, Communication, & Automation (ICACCA) (Spring).

[12] Manjeshwar A., Agrawal D.P. TEEN: A routing protocol for enhanced efficiency in wireless sensor networks. Proceedings of the 15th International Parallel and Distributed Processing Symposium.

[13] Kodali R.K., Kiran A. V.S., Bhandari S., Boppana L. Energy efficient m- level LEACH protocol. Proceedings of the International Conference on Advances in Computing, Communications and Informatics (ICACCI).

[14] Lindsey S., Raghavendra C.S. PEGASIS: Power-efficient gathering in sensor information systems. Proceedings of the IEEE Aerospace Conference.

[15] Truong A.S.-C.a.H.L. MQTT For Sensor Networks (MQTT-SN) Protocol Specification. http://mqtt.org/2013/12/mqtt-for-sensor-networks-mqtt-sn.

[16] Bormann C., Lemay S., Tschofenig H., Hartke K., Silverajan B., Raymor B. CoAP (Constrained Application Protocol) over TCP, TLS, and WebSockets. In RFC 8323, 10.17487/RFC8323. https://tools.ietf.org/id/draft-ietf-core-coap-tcp-tls-11.html.

[17] Che X., Wells I., Dickers G., Kear P., Gong X. (2010). Re-evaluation of RF electromagnetic communication in underwater sensor networks. IEEE Commun. Mag..

[18] Ryecroft S.P., Shaw A., Fergus P., Kot P., Muradov M., Moody A., Conroy L. Requirements of an underwater sensor-networking platform for environmental monitoring. Proceedings of the Developments in Esystems Engineering.

[19] Lloret J., Sendra S., Ardid M., Rodrigues J.J.P.C. (2012). Underwater Wireless Sensor Communications in the 2.4 GHz ISM Frequency Band. Sensors.

[20] Rubino E.M., Centelles D., Sales J., Marti J.V., Marin R., Sanz P.J. (2015). Wireless Image Compression and Transmission for Underwater Robotic Applications?. IFAC-Papers Online.

[21] Shaw A., Al-Shamma’a A.i., Wylie S.R., Toal D. Experimental Investigations of Electromagnetic Wave Propagation in Seawater. Proceedings of the 2006 European Microwave Conference.

[22] Abdou A.A., Shaw A., Mason A., Al-Shamma’a A., Cullen J., Wylie S., Diallo M. (2013). A matched Bow-tie antenna at 433MHz for use in underwater wireless sensor networks. J. Phys.: Conf. Ser..

[23] Abdou A., Shaw A., Mason A., Al-Shamma A., Cullen J.D., Wylie S. Electromagnetic (EM) wave propagation for the development of an underwater Wireless Sensor Network (WSN). Proceedings of the 2011 IEEE Sensors.

[24] Warren L., Stutzman G.A.T. (2012). Antenna Theory and Design.

[25] Bluecomm BlueComm Underwater Optical Communication-Sonardyne. https://www.sonardyne.com/product/bluecomm-underwater-optical-communication-system/.

[26] Marine T. Teledny Marine 900 series. http://www.teledynemarine.com/acoustic-modems.

[27] Codarin A., Wysocki L.E., Ladich F., Picciulin M. (2009). Effects of ambient and boat noise on hearing and communication in three fish species living in a marine protected area (Miramare, Italy). Mar. Pollut. Bull..

[28] Peng C., Zhao X., Liu G. (2015). Noise in the Sea and Its Impacts on Marine Organisms. Int. J. Environ. Res. Public Health.

[29] Weik M.H. (2001). Nyquist theorem. Computer Science and Communications Dictionary.

[30] Shannon C.E. (1949). Communication in the Presence of Noise. Proc. IRE.

[31] Hartley R.V.L. (1928). Transmission of Information. Bell Syst. Tech. J..

